# Effects of Aerobic Exercise on Cardiorespiratory Fitness and Cardiovascular Risk Factors in Long-Term Breast Cancer Survivors: A Randomized Controlled Trial

**DOI:** 10.1016/j.jaccao.2025.04.006

**Published:** 2025-06-17

**Authors:** Sara H. Johansen, Mali Sæter, Sebastian I. Sarvari, Kristin V. Reinertsen, Elisabeth Edvardsen, Torbjørn Wisløff, Jessica M. Scott, May Grydeland, Truls Raastad, Jostein Hallén, Cecilie E. Kiserud, Hanne C. Lie, Paul A. Solberg, Kristina Hermann Haugaa, Johanne S.S. Jensen, Line H. Vatningen, Lene Thorsen, Tormod S. Nilsen

**Affiliations:** a Department of Physical Performance, The Norwegian School of Sport Sciences, Oslo, Norway; b Institute of Clinical Medicine, Faculty of Medicine, University of Oslo, Oslo, Norway; c ProCardio Center for Innovation, Department of Cardiology, Oslo University Hospital, Oslo, Norway; d Department of Oncology, Oslo University Hospital, Oslo, Norway; e Department of Pulmonary Medicine, Oslo University Hospital, Oslo, Norway; f Health Services Research Unit, Akershus University Hospital, Lørenskog, Norway; g Memorial Sloan Kettering Cancer Center, New York, New York, USA; h Weill Cornell Medical College, New York, New York, USA; i Department of Behavioural Medicine, University of Oslo, Oslo, Norway; j Norwegian Olympic and Paralympic Committee and Confederation of Sports, Oslo, Norway; k Department for Clinical Service, Division of Cancer Medicine, Oslo University Hospital, Oslo, Norway

**Keywords:** breast cancer survivors, breast cancer, cancer survivorship, cardiorespiratory fitness, cardiovascular risk factors, exercise oncology, exercise therapy

## Abstract

**Background:**

Cancer treatment may impair physiological adaptations to exercise therapy, yet no study has directly compared exercise effects between cancer survivors and cancer-naive control subjects.

**Objectives:**

This study sought to examine the effects of aerobic exercise in anthracycline-treated long-term survivors of breast cancer (BCS) and to compare the effects to cancer-naive women.

**Methods:**

The CAUSE (CArdiovascUlar Survivors Exercise) trial was a 2-arm randomized controlled trial in which long-term BCS were assigned to thrice-weekly nonlinear aerobic exercise for 5 months (BCS exercise) or usual care (BCS usual care). A third group of similarly aged cancer-naive women completed the same exercise intervention. The primary outcome was cardiorespiratory fitness (CRF) (measured as Vo_2peak_). Secondary outcomes included cardiovascular risk factors (cardiometabolic biomarkers and body composition) and patient-reported outcomes (subjective vitality and life satisfaction).

**Results:**

Between October 2020 and February 2023, 140 BCS (aged 59.0 ± 6.4 years; 11 ± 1 years after treatment) and 69 cancer-naive women (aged 57.8 ± 4.9 years) were enrolled. From baseline to post-exercise intervention, Vo_2peak_ increased by 1.2 ± 2.6 mL·kg^−1^·min^−1^ in the BCS exercise, by 0.01 ± 2.5 mL·kg^−1^·min^−1^ in the BCS usual care group (mean difference 1.3; 95% confidence interval [CI]: 0.5-2.1; *P* = 0.002), and by 2.6 ± 2.5 mL·kg^−1^·min^−1^ in non-cancer subjects (BCS exercise vs non-cancer subjects: mean difference −1.4; 95% CI: −2.2 to −0.5; *P* = 0.003). No changes in cardiovascular risk factors were observed. Compared with BCS usual care, the BCS exercise group reported improved subjective vitality (mean difference 2.56; 95% CI: 1.22-3.90; *P* < 0.001) and satisfaction with life (mean difference 1.68; 95% CI: 0.43-2.93; *P* = 0.009).

**Conclusions:**

Although aerobic exercise improves CRF in anthracycline-treated long-term BCS, the response was less than one-half that observed in cancer-naive subjects.

Although advances in breast cancer detection and treatment have improved survival rates, adjuvant chemotherapy is associated with late toxicity.^1^ Cardiorespiratory fitness (CRF), assessed by peak oxygen consumption (Vo_2peak_), is an integrative measure of global cardiovascular capacity^2^^,^^3^ and is a reliable marker of overall health.^4^ In cancer survivors, poor CRF is associated with attendant adverse outcomes, including greater symptom burden^5^ and increased treatment-related cardiovascular risk factors.^6^ Vo_2peak_ typically declines by approximately 10% after systemic treatment,^7^ a reduction equivalent to a decade of healthy aging occurring within the 3 to 6 months of treatment.^8^ Importantly, this decline may persist even after treatment ends, elevating the risk of morbidity and premature mortality in cancer survivors.^9^, ^10^, ^11^

Aerobic exercise is widely recognized as an effective strategy to mitigate cardiovascular events in non-oncological populations,^12^ and has been shown to improve CRF through exercise therapy both during^13^, ^14^, ^15^ and after treatment in early-stage survivors of breast cancer (BCS).^15^^,^^16^ However, prior studies in BCS have reported only modest improvements in CRF with exercise therapy,^15^^,^^16^ accompanied by substantial interindividual heterogeneity within intervention groups.^16^^,^^17^ This has led to the suggestion that prior cancer treatment may impair physiological adaptations to exercise therapy.^15^^,^^16^ Given the limited research in long-term BCS (ie, more than five years after treatment completion), it remains unclear whether these impaired exercise responses persist throughout survivorship.

Moreover, to our knowledge, no prior study has compared exercise responses between BCS and matched cancer-naive women. Against this background, this randomized controlled trial aimed to: 1) evaluate the effects of aerobic exercise on CRF and other cardiovascular risk factors in long-term BCS; and 2) compare these effects with those in cancer-naive women.

## Methods

### Design and study sample

The CAUSE (CArdiovascUlar Survivors Exercise) trial was a 2-arm, phase II randomized controlled trial at the Norwegian School of Sport Sciences (Oslo, Norway), comparing aerobic exercise (BCS exercise) with usual care (BCS usual care) in BCS.^18^ In addition, similarly aged cancer-naive women (non-cancer subjects) were included. The study was conducted in accordance with the Declaration of Helsinki, approved by the Regional Committees for Medical and Health Research Ethics (REC; 2019/1318), and preregistered at ClinicalTrials.gov (NCT04307407). The CAUSE trial followed the CONSORT guidelines for non-pharmacological interventions (Supplemental Section 1).

BCS were identified through the Cancer Registry of Norway and invited to participate by postal mail. Potential participants were screened for eligibility by phone. Inclusion criteria were female survivors of stage Il-III breast cancer diagnosed between 2008 and 2012, age ≤60 years at diagnosis, and prior treatment with epirubicin. Exclusion criteria included self-reported engagement in ≥90 minutes of exercise per week; previous treatment with trastuzumab; recurrent breast cancer or other malignancies (except basal cell carcinoma); pacemaker therapy; prior major cardiac surgery; recent or uncontrolled cardiovascular disease; health conditions that, by self-assessment, would limit adherence to the study protocol; medical contraindications to exercise; or participation in other exercise trials.

Participants in the non-cancer control group were recruited through digital news media advertisements and screened for eligibility by phone. Inclusion criteria were women with no history of malignancy and an age range similar to that of the BCS cohort (typically 40 to 70 years). All other exclusion criteria were the same as those applied to the BCS.

### Randomization and blinding

Following baseline assessments, BCS were randomly assigned in a 1:1 ratio to either aerobic exercise or usual care, without additional stratification factors. A random sequence of study IDs was generated before enrollment using random number generator. The randomization sequence was concealed from assessment personnel until the completion of the baseline assessments. Both study participants and assessment personnel were aware of group assignments during postintervention assessments but remained blinded to baseline results.

### Exercise intervention

A detailed description of the exercise intervention has been published previously.^18^ Briefly, the intervention consisted of supervised treadmill walking or running sessions, performed 3 times per week over 5 months. Using a nonlinear progression model, the exercise program included both continuous sessions at low-to-moderate intensity (25-40 minutes at 60%-85% of peak heart rate [HR_peak_]) and high-intensity interval sessions (16-32 minutes at 85%-97% of HR_peak_) (Supplemental Section 2, Supplemental Figure 1). The exercise prescription was individually tailored to each participant’s cardiopulmonary exercise test (CPET) result (HR_peak_) and adjusted based on ratings of perceived exertion using the Borg Scale.^19^ Instructors documented any deviations from the prescribed exercise and recorded reasons for those deviations.^20^ Participants in the usual care group were encouraged to maintain their activity level during the intervention period but did not receive any exercise follow-up.

### Outcomes

All measurements were performed by a cardiologist and qualified exercise physiologists at baseline (1 to 2 weeks before the exercise intervention) and within 1 week after the 5-month intervention period (S.H.J., M.S., J.S.S.J., L.H.V.).

#### Primary Outcome: Cardiorespiratory fitness

CRF was assessed as Vo_2peak_, measured via maximal CPET using uphill treadmill walking (RL2700E, Rodby) following a modified Balke-protocol^21^ consistent with the reference population.^22^ The test began at a speed of 4.0 to 4.8 km/h, with the incline increasing by 2% every minute. After reaching the maximum incline of 20%, the speed increased by 0.4 km/h every minute until exhaustion. Expired gases were measured breath-by-breath using a gas- and volume-calibrated metabolic chart (Oxycon Pro, Jaeger) and reported as 30-second averages. Oxygen saturation, ECG, and arterial blood pressure were continuously monitored. The rating of perceived exertion was assessed using the Borgs_6-20_ scale,^19^ and blood lactate concentration was measured from a capillary blood sample collected 60 seconds after termination (Lactate Scout 4, EKF Diagnostic) to evaluate the degree of effort (Supplemental Section 3, Supplemental Table 1).

#### Secondary Outcomes and Covariates: Cardiovascular Risk Factors and Patient-Reported Outcomes

Resting arterial blood pressure was measured in the supine position using an electronic sphygmomanometer (Welch Allyn ProBP 2400). Height and weight were measured using a stadiometer (SECA 213), and body mass index (BMI) was calculated accordingly. Body composition was assessed using whole-body dual x-ray absorptiometry (Lunar iDXA, GE Healthcare) in an overnight fasted state and analyzed with enCORE Software 14.10 (GE HealthCare). Fasting blood samples were analyzed at Fürst Laboratories following established procedures. Self-reported physical activity was assessed using a modified version of the Godin-Shepard Leisure-Time Physical Activity Questionnaire,^23^ which captures the average weekly frequency and duration of mild, moderate, and vigorous physical activity. Participants’ feelings of aliveness and energy were assessed using the 5-item Subjective Vitality Scale (SVS),^24^ and life satisfaction was assessed using the 5-item Satisfaction With Life Scale (SWLS)^25^ (Supplemental Section 3).

#### Background variables

Sociodemographic variables (living status and education level) and daily smoking habits were assessed using items from the HUNT4 study.^26^ Current medications, including antihypertensive, lipid-lowering, and glucose-lowering agents, and beta-blockers, were obtained from case histories and medical records. Cancer-related variables—age at diagnosis, time since epirubicin discontinuation and dosage, and treatment modality—were obtained from medical records.

#### Tolerability, adherence, and safety

Tolerability of the intervention was assessed based on session attendance, exercise dose modifications, and loss to follow-up.^20^ Adherence to the exercise prescription was measured using relative dose intensity. Interruptions in the intervention were defined as missing three or more consecutive sessions. Exercise dosage (planned and completed) for all exercise sessions was calculated using a modified version of Training Impulse (TRIMP), which incorporated heart rate, duration of time spent in the prescribed training zone (in minutes), and a weighting factor to account for session type (with higher intensity sessions receiving greater weighting due to shorter duration). Safety was evaluated by recording the type and frequency of adverse events and serious adverse events occurring during both the exercise test and the aerobic training sessions.

### Statistical analysis

Given the limited literature available at the time of the trial design, the sample size calculation was based on the primary outcome, Vo_2peak_, assuming a mean change of 3.6 mL·kg^−1^·min^−1^ and a standard deviation of 7.2 mL·kg^−1^·min^−1^, as reported in a similar intervention by Adams et al.^27^ Using a significance level of 5% and higher-than-usual power of 90%—to account for relying on a single published study—63 BCS were required in each group. Allowing for a 10% dropout rate, 70 BCS were enrolled in each group, with an equal number included in the non-cancer control group.

Continuous data are presented as mean ± SD or median with 25th-75th percentiles (Q1-Q3), and categorical data as count (percentage). Primary analyses followed the intention-to-treat principle, assuming no change (ie, change = 0) for participants with missing follow-up data. Between-group differences in postintervention values (BCS exercise vs BCS usual care) were evaluated using analysis of covariance, adjusting for baseline values of the respective outcome. Results are reported as least squares mean differences (LSMDs) with 95% CIs.

Additional analyses were conducted to compare BCS exercise with non-cancer subjects. The normality of residuals was assessed by visual inspection of histograms and Q–Q plots. Baseline characteristics between BCS and non-cancer subjects were compared using Student’s *t* test for normally distributed continuous variables, Mann-Whitney *U* test for all other continuous variables, chi-square test for dichotomous data with >5 expected cell counts per cell, and Fisher's exact test otherwise. Changes from pre- to postintervention were evaluated using analysis of covariance, adjusting for age, BMI, and self-reported physical activity (minutes of moderate and vigorous physical activity).

Additional per-protocol analyses included only participants in the exercise groups who adhered to >70% of the prescribed exercise regimen, and participants in the BCS usual care group whose self-reported moderate-to-vigorous physical activity changed by <90 minutes from pre- to postintervention.

All statistical tests were 2-tailed, with a significance level of *P* < 0.05. Analyses were conducted using IBM SPSS Statistics (Version 28.0.1.0, IBM Corp).

## Results

**Figure 1:**
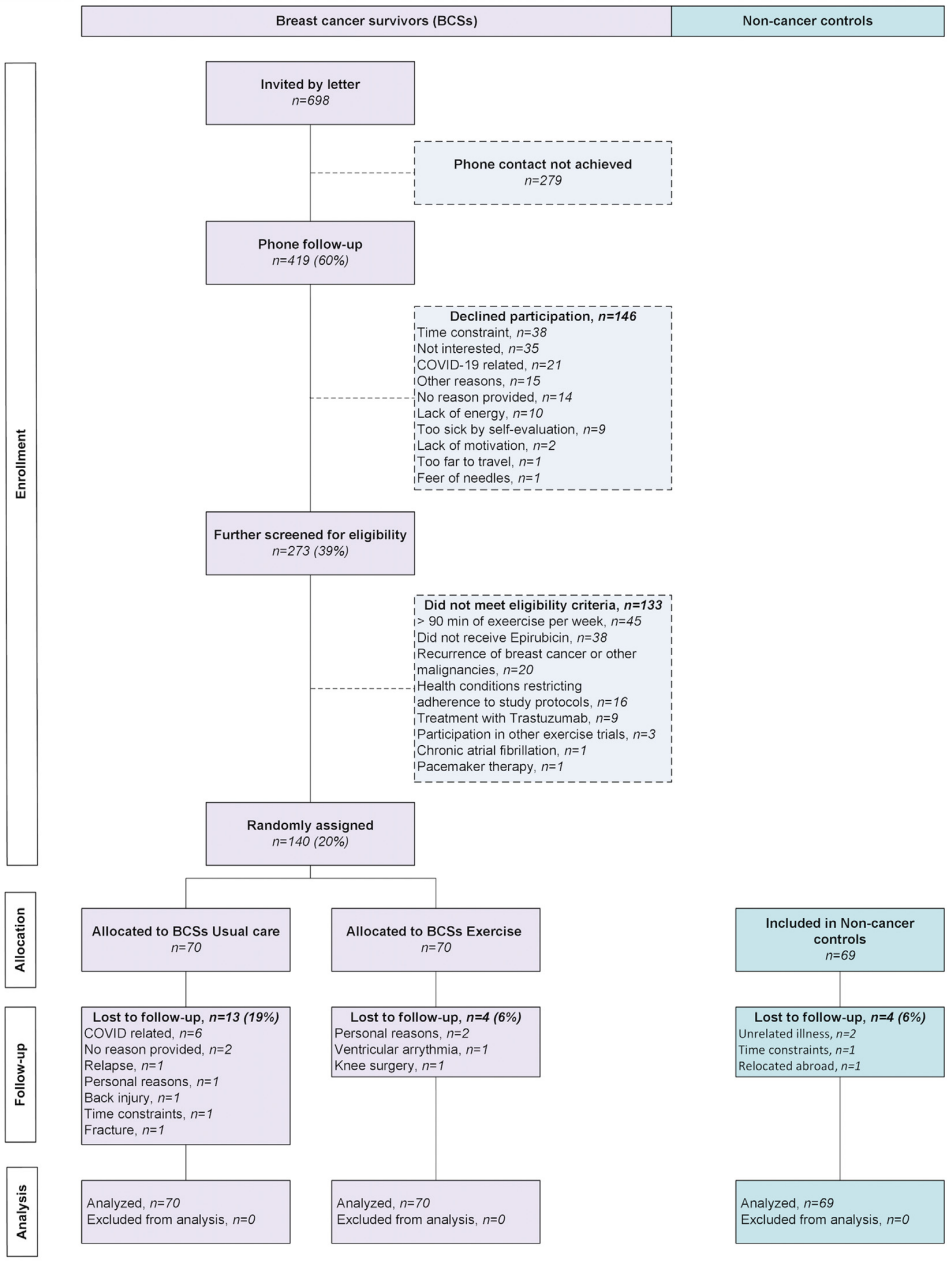
CONSORT Flow Diagram for Participant Inclusion Flow diagram showing the inclusion process for participants, following CONSORT guidelines. The figure details the number of participants invited, screened, excluded, and allocated to the exercise and control groups, along with reasons for loss to follow-up.

Between October 2020 and August 2022, 140 BCS (mean age 59.0 ± 6.4 years) were enrolled (Figure 1). The average time since epirubicin treatment was 11 ± 1 years, with a median cumulative dose of 357 mg·m^−2^ (Q1-Q3: 243-366) (Table 1). Seventy cancer-naive women (mean age 57.8 ± 4.9 years) were included in the non-cancer control group; 1 participant withdrew before the start of the study (Figure 1). Pre-randomization Vo_2peak_ was 27.6 ± 5.4 mL·kg^−1^·min^−1^ in BCS and 27.1 ± 5.4 mL·kg^−1^·min^−1^ in non-cancer subjects, corresponding to 91% and 89% of predicted values,^22^ respectively (*P* = 0.52). Baseline demographics were similar between groups, except for education level (*P* = 0.035) (Table 1).

**Table 1:** Baseline Characteristics of Participants

	Total BCS (N = 140)	BCS Exercise (n = 70)	BCS Usual Care (n = 70)	Non-Cancer Control Subjects (n = 69)	*P* Value^a^
Sociodemographic					
Age at survey, y	59.0 ± 6.4	58.6 ± 6.0	59.4 ± 6.8	57.8 ± 4.9	0.16
Living with partner					0.31
Yes	99 (70.7)	51 (72.9)	48 (68.6)	44 (63.7)	
No	41 (29.3)	19 (27.1)	22 (31.4)	25 (36.2)	
Education level >13 y					0.035
Yes	93 (66.4)	44 (62.9)	49 (70.0)	56 (81.2)	
No	46 (32.9)	26 (37.1)	20 (28.6)	13 (18.8)	
Lifestyle and health					
Height, cm	168.0 ± 5.8	168.0 ± 5.4	168.1 ± 6.3	167.9 ± 6.1	0.91
Body mass, kg	76.0 ± 13.5	75.5 ± 13.0	76.5 ± 14.1	79.3 ± 14.5	0.11
Body mass index, kg/m^2^	26.9 ± 4.5	26.7 ± 4.4	27.1 ± 4.6	28.1 ± 5.0	0.077
Daily smokers	6 (4.2)	3 (4.3)	3 (4.3)	2 (2.9)	0.72
Meet WHO physical activity recommendation^b^	30 (21.4)	14 (20.0)	16 (22.9)	11 (15.9)	0.40
Antihypertensive medication	18 (12.9)	7 (10.0)	11 (15.7)	12 (17.4)	0.38
Lipid-lowering medication	15 (10.7)	6 (8.6)	9 (12.9)	9 (13.0)	0.62
Glucose-lowering medication	5 (3.6)	2 (2.9)	3 (4.3)	2 (2.9)	>0.99
Beta-blocker	5 (3.6)	2 (2.9)	3 (4.3)	0	0.17
NT-proBNP, ng/L	70 [40-115]	74 [33-139]	67 [46-111]	68 [39-110]	0.58
Troponin T, ng/L	5 [4-6]	5 [4-6]	5 [4-6]	5 [4-6]	0.52
Cancer-related variables					
Age at diagnosis, y	48.0 ± 6.2	47 ± 5.9	48 ± 7.0		
Years since epirubicin discontinuation, y	11 ± 1, range: 8-14	11 ± 1, range: 8-14	11 ± 1, range: 8-13		
Hormone receptor-positive	109 (77.9)	53 (75.7)	56 (80.0)		
Cumulative epirubicin dose, mg·m^−2^^c^^,^^d^	357 [243-366] range: 227-598	358 [247-365] range: 227-598	353 [241-367] range: 229-597		
<310	49 (35.0)	21 (30.0)	28 (40)		
310 to <500	88 (62.9)	48 (68.6)	40 (57.1)		
>500	2 (1.4)	1 (1.4)	1 (1.4)		
Taxane therapy^c^	69 (49.3)	32 (45.7)	37 (52.9)		
Radiotherapy	119 (85.0)	56 (80.0)	63 (90.0)		
Left-sided radiotherapy	72 (51.4)	41 (58.6)	31 (44.2)		
Endocrine therapy	110 (78.6)	54 (78.6)	56 (80.0)		
Tamoxifen only	54 (38.6)	28 (40.0)	26 (37.1)		
Aromatase inhibitor only	20 (14.3)	10 (14.3)	10 (14.3)		
Tamoxifen+aromatase inhibitor	36 (25.7)	16 (22.9)	20 (28.6)		

Values are mean ± SD, n (%),or median [Q1-Q3]. *P* values are derived from Student’s *t*-test for normally distributed continuous variables, Mann-Whitney *U* test for non-normally distributed continuous variables, and chi-square test or Fisher's exact test for categorical variables.

NT-proBNP = N-terminal pro–B-type natriuretic peptide; WHO = World Health Organization.

a Comparison between total breast cancer survivors (BCS) and non-cancer control subjects.

b >150 minutes of moderate physical activity or >75 minutes of vigorous physical activity per week, or an equivalent combination.

c Details on chemotherapy regimen were missing from 1 patient.

d Administered in combination with 5-fluorouracil and cyclophosphamide.

### Tolerability, adherence, and safety

Loss to follow-up was 5.7% (n = 4) in BCS exercise, 18.6% (n = 13) in BCS usual care, and 5.8% (n = 4) in non-cancer subjects. Mean intervention attendance was 78.5% in BCS exercise and 79.9% in non-cancer subjects (*P* = 0.55) (Supplemental Section 4). Exercise interruptions—defined as missing 3 or more consecutive sessions—were reported by 75.0% of BCS exercise and 71.0% of non-cancer subjects, with mean relative dose intensity of 72.3% and 73.3%, respectively. One adverse event occurred during baseline assessments: a participant stumbled off the treadmill after CPET termination, resulting in a wrist fracture. This was promptly managed by the study’s medical team and delayed the start of her intervention. Additionally, unrelated ankle fractures occurred during the intervention in 1 participant in the BCS exercise group and 1 in the usual care group, resulting in exercise modification and dropout, respectively.

### Effect of exercise therapy on CRF and cardiovascular risk factors in BCS

Vo_2peak_ increased by 1.2 ± 2.6 mL·kg^−1^·min^−1^ in BCS exercise compared with 0.01 ± 2.5 mL·kg^−1^·min^−1^ in BCS usual care (LSMD 1.3; 95% CI: 0.5-2.1; *P =* 0.002). In absolute terms, Vo_2peak_ increased by 0.08 ± 0.19 L/min in BCS exercise, compared with a decrease of −0.01 ± 0.19 L/min in BCS usual care (LSMD 0.1; 95% CI: 0.03-0.16; *P =* 0.003). The oxygen pulse increased by 0.66 ± 1.08 mL/beat^−1^ in BCS exercise and 0.03 ± 1.14 mL/beat^−1^ in BCS usual care (LSMD 0.62; 95% CI: 0.26-0.98; *P* < 0.001). No significant changes were observed in cardiovascular risk factors, although a small but statistically significant increase in HbA_1c_ (glycated hemoglobin) was observed in BCS exercise (LSMD 0.68; 05% CI: 0.07-1.29; *P =* 0.030) (Table 2).

**Table 2:** Effects of Aerobic Exercise on Cardiorespiratory Fitness, Cardiovascular Risk Factors, and Patient-Reported Outcomes in BCS Exercise Compared With BCS Usual Care and Non-Cancer Control Subjects

	BCS Exercise			BCS Usual Care			Non-Cancer Control Subjects			BCS Exercise vs Usual Care^a^		BCS Exercise vs Non-Cancer Control Subjects^b^	
	n	Pre (n = 70)	Post (n = 70)	n	Pre (n = 70)	Post (n = 70)	n	Pre (n = 69)	Post (n = 69)	Least Squares Mean Difference (95% CI)	*P* Value	Least Squares Mean Difference (95% CI)	*P* Value
Cardiorespiratory fitness													
Vo_2peak_, L·min^−1^	70	2.07 ± 0.34	2.16 ± 0.34	70	2.05 ± 0.40	2.04 ± 0.39	69	2.10 ± 0.34	2.28 ± 0.36	0.10 (0.03 to 0.16)	0.003	−0.09 (−0.15 to −0.02)	0.007
Vo_2peak_, mL·kg^−1^·min^−1^	70	28.0 ± 5.5	29.3 ± 5.4	70	27.2 ± 5.3	27.2 ± 5.2	69	27.1 ± 5.4	29.7 ± 5.9	1.3 (0.5 to 2.1)	0.002	−1.4 (−2.2 to −0.5)	0.003
Vo_2_ at VT, L·min^−1^	69	1.71 ± 0.34	1.72 ± 0.35	69	1.70 ± 0.37	1.67 ± 0.34	68	1.73 ± 0.36	1.80 ± 0.34	0.04 (−0.04 to 0.12)	0.27	−0.05 (−0.14 to 0.03)	0.21
HR_peak_, beats·min^−1^	69	175 ± 12	172 ± 13	70	174 ± 11	173 ± 11	69	172 ± 10	171 ± 8	−0.84 (−2.79 to 1.11)	0.40	−0.97 (−3.34 to 1.41)	0.42
O_2-_pulse, mL·beat^−1^	69	11.9 ± 1.77	12.5 ± 1.86	70	11.8 ± 2.36	11.9 ± 2.24	69	12.5 ± 1.86	13.4 ± 1.89	0.62 (0.26 to 0.98)	<0.001	−0.41 (−0.76 to −0.05)	0.028
Peak systolic BP, mm Hg	38	196 ± 22	194 ± 21	40	201 ± 21	194 ± 23	54	187 (24)	192 ± 26	1.8 (−7.0 to 10.5)	0.69	−2.80 (−12.80 to 7.21)	0.58
Peak diastolic BP, mm Hg	38	79 ± 17	75 ± 17	40	78 ± 17	82 ± 14	54	72 ± 15	76 ± 16	−7.7 (−12.3 to −2.5)	0.005	−8.67 (−15.67 to −1.86)	0.013
VE_peak_ L·min^−1^	70	77 ± 15	84 ± 16	70	77 ± 17	78 ± 16	69	80 ± 15	85 ± 15	6.5 (3.3 to 9.7)	<0.001	2.0 (−1.5 to 5.5)	0.26
VE/VCo_2_ slope	70	27 ± 4	28 ± 4	69	27 ± 4	27 ± 4	67	27 ± 4	27 ± 3	0.3 (−0.7 to 1.4)	0.56	1.0 (−0.2 to 2.2)	0.10
Spo_2_ at max effort, %	59	95 ± 2	94 ± 3	62	94 ± 3	94 ± 3	57	95 ± 3	94 ± 3	−0.5 (−1.2 to 0.2)	0.12	−0.1 (−0.8 to 0.6)	0.80
RER, VCo_2_·Vo_2_^−1^	70	1.19 ± 0.09	1.21 ± 0.08	70	1.21 ± 0.08	1.20 ± 0.08	69	1.23 ± 0.08	1.23 ± 0.06	0.02 (0.00 to 0.04)	0.02	0.03 (0.00 to 0.05)	0.032
Blood lactate concentration, mmol/L	64	8.8 ± 3.0	8.6 ± 2.8	63	8.8 ± 2.5	8.2 ± 2.4	57	8.4 ± 2.10	9.3 ± 7.1	0.44 (−0.19 to 1.08)	0.17	−1.31 (−3.21 to 0.59)	0.18
Borgs scale_6-20_	66	17 ± 1	18 ± 1	63	17 ± 2	17 ± 2	65	17 ± 1	18 ± 1	0.50 (0.12 to 0.87)	0.010	−0.35 (−0.79 to 0.09)	0.12
Cardiovascular risk factors													
Self-reported moderate-to-vigorous physical activity, min/wk	70	94.7 ± 132.8	148.6 ± 138.5	70	97.2 ± 153.4	112.5 ± 139.6	69	77.1 ± 156.6	137.3 ± 100.4	37.0 (−17.9 to 65.9)	0.09	3.4 (−33.7 to 40.6)	0.86
Systolic blood pressure, mm Hg	70	135 ± 18	137 ± 17	70	135 ± 18	135 ± 18	69	138 ± 19	137 ± 19	0.72 (−2.30 to 3.75)	0.72	1.26 (−2.12 to 4.64)	0.46
Diastolic blood pressure, mm Hg	70	83 ± 8	82 ± 9	70	82 ± 7	81 ± 8	69	81 ± 8	80 ± 8	−0.13 (−2.01 to 1.76)	0.90	0.05 (−1.96 to 2.06)	0.96
Fasting glucose, mmol·L^−1^	69	5.26 ± 0.66	5.21 ± 0.77	64	5.22 ± 0.93	5.26 ± 1.05	63	5.24 ± 0.75	5.20 ± 0.79	−0.09 (−0.25 to 0.07)	0.29	−0.04 (−0.18 to 0.11)	0.61
HbA_1c_, mmol·mol^−1^	68	37.0 ± 3.8	37.7 ± 4.05	64	38.6 ± 4.89	38.0 ± 4.65	67	37.3 ± 4.90	37.6 ± 4.43	0.68 (0.07 to 1.29)	0.030	0.01 (−0.59 to 0.58)	0.99
Insulin, pmol·L^−1^	64	60.1 ± 35.1	56.4 ± 36.5	64	64.1 ± 74.4	62.0 ± 72.8	61	54.3 ± 30.1	56.9 ± 34.4	−2.01 (−10.4 to 6.4)	0.64	−5.52 (−11.43 to 0.39)	0.067
Total cholesterol, mmol·L^−1^	69	5.84 ± 0.96	5.80 ± 0.98	65	6.00 ± 0.90	5.94 ± 0.92	68	5.63 ± 1.02	5.48 ± 1.13	−0.01 (−0.21 to 0.19)	0.91	0.17 (−0.05 to 0.39)	0.12
HDL-C, mmol·L^−1^	69	1.78 ± 0.46	1.80 ± 0.46	66	1.81 ± 0.42	1.79 ± 0.42	68	1.66 ± 0.36	1.69 ± 0.38	0.04 (−0.03 to 0.12)	0.24	0.00 (−0.07 to 0.06)	0.92
LDL-C, mmol·L^−1^	69	3.77 ± 0.93	3.68 ± 0.98	65	3.96 ± 0.92	3.95 ± 0.91	68	3.74 ± 1.08	3.71 ± 1.18	−0.12 (−0.32 to 0.09)	0.25	0.01 (−0.22 to 0.24)	0.91
Triglycerides, mmol·L^−1^	69	1.26 ± 0.65	1.31 ± 0.67	65	1.24 ± 0.75	1.23 ± 0.72	62	1.23 ± 0.51	1.16 ± 0.41	0.00 (−0.14 to 0.14)	0.95	0.05 (−0.07 to 0.17)	0.43
hsCRP, mg·L^−1^	69	2.41 ± 4.92	2.47 ± 4.67	65	2.23 ± 4.20	1.95 ± 3.69	65	2.56 ± 4.21	2.33 ± 2.78	0.16 (−1.09 to 1.41)	0.80	0.66 (−0.64 to 1.96)	0.32
Total fat mass, kg	70	30.4 ± 9.29	29.8 ± 9.30	70	31.2 ± 9.52	31.2 ± 9.40	69	33.5 ± 11.3	32.1 ± 10.5	−0.38 (−1.03 to 0.26)	0.24	0.74 (−0.26 to 1.74)	0.14
Fat percentage, %	70	39 ± 6	39 ± 6	70	40 ± 6	40 ± 6	69	41 ± 7	40 ± 7	−0.46 (−0.99 to 0.06)	0.08	0.30 (−0.26 to 0.87)	0.29
Total lean body mass, kg	70	42.6 ± 4.5	42.8 ± 4.43	70	42.5 ± 5.4	42.5 ± 5.3	69	43.3 ± 4.36	43.5 ± 4.41	0.11 (−0.22 to 0.43)	0.52	−0.04 (−0.43 to 0.36)	0.85
Patient-reported outcomes													
SVS total score	69	23.6 ± 6.8	26.7 ± 6.7	68	24.2 ± 6.5	24.6 ± 5.9	69	25.9 ± 6.4	27.9 ± 6.4	2.56 (1.22 to 3.90)	<0.001	0.98 (−0.61 to 2.58)	0.23
SWLS total score	69	24.8 ± 5.9	27.3 ± 6.2	68	25.3 ± 5.7	26.1 ± 5.8	69	26.5 ± 6.4	27.3 ± 7.2	1.68 (0.43 to 2.93)	0.009	1.71 (0.26 to 2.16)	0.02

Values are mean ± SD. *P* values are derived from analysis of covariance.

BCS = breast cancer survivor; BP = blood pressure; CRP = C-reactive protein; HbA_1c_ = glycated hemoglobin; HDL-C = high-density lipoprotein cholesterol; HR_peak_ = peak heart rate; LDL-C = low-density lipoprotein cholesterol; mmHg = millimeters of mercury; o_2_-pulse = oxygen pulse; RER = respiratory exchange ratio; Spo_2_ = oxygen saturation; SVS = Subjective Vitality Scale; SWLS = Satisfaction With Life Scale; VE/VCo_2_ = ventilatory equivalent for carbon dioxide output; VE_peak_ = peak minute ventilation; Vo_2peak_ = peak oxygen consumption; VT = ventilatory threshold.

a Analyses adjusted for baseline values of the respective outcome variable.

b Analyses adjusted for age, body mass index, and baseline self-reported moderate-to-vigorous physical activity.

### Response to exercise therapy in BCS compared with non-cancer subjects

**Figure 2:**
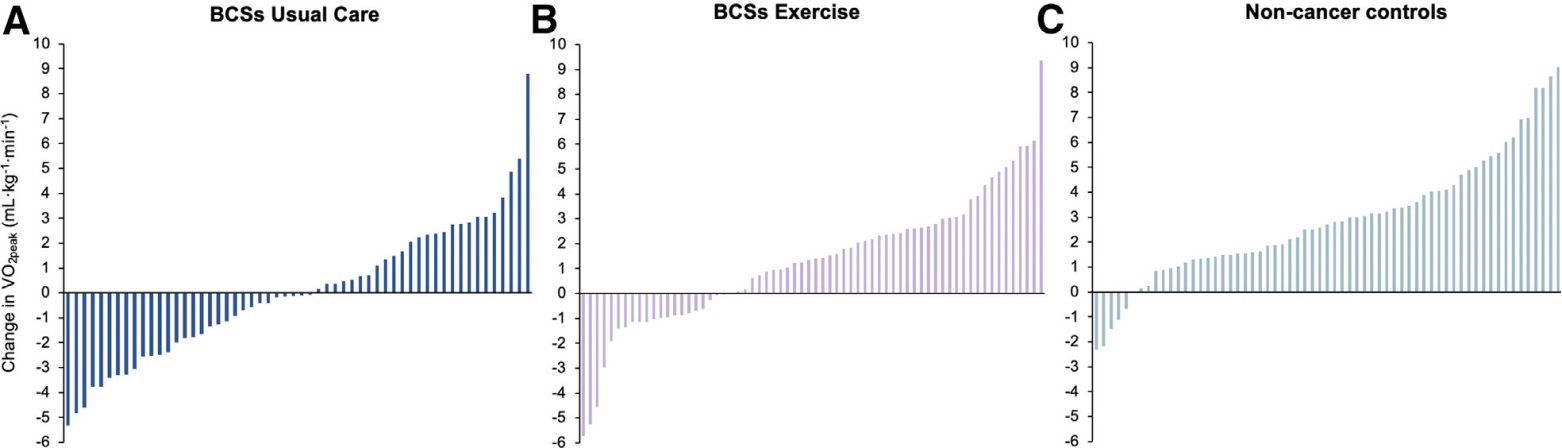
Individual Changes in Vo_2peak_ After 5 Months of Aerobic Exercise Waterfall plots illustrate individual changes in peak oxygen consumption (Vo_2peak_) (mL·kg^−1^·min^−1^) after 5 months of aerobic exercise. Each bar represents 1 participant. (A) Survivors of breast cancer (BCS) in the usual care group, (B) BCS in the exercise group, and (C) non-cancer subjects; individual VO_2peak_ changes are shown for each participant.

The improvement in CRF from pre- to postintervention was significantly smaller in BCS exercise than in non-cancer subjects, with Vo_2peak_ increasing by 5.1% (1.2 ± 2.6 mL·kg^−1^·min^−1^) and 10.3% (2.6 ± 2.5 mL·kg^−1^·min^−1^), respectively (LSMD −1.4; 95% CI: −2.2 to −0.5; *P =* 0.003) (Table 2). The change in Vo_2peak_ ranged from −5.7 to 9.3 mL·kg^−1^·min^−1^ in BCS exercise and −2.3 to 9.0 mL·kg^−1^·min^−1^ in non-cancer subjects (Figure 2). No significant differences in changes to cardiovascular risk factors were observed between groups (Table 2).

### Subjective Vitality and Life Satisfaction

At baseline, BCS (n = 140) reported lower vitality (23.9 ± 6.6 vs 25.9 ± 6.4; *P =* 0.042) and life satisfaction (25.1 ± 5.8 vs 26.5 ± 6.4; *P =* 0.13) compared with non-cancer subjects. After the intervention, SVS scores increased by 3.1 ± 4.3 points in BCS exercise, 0.4 ± 4.3 points in BCS usual care (LSMD 2.6; 95% CI: 1.2-3.9; *P* < 0.001), and 2.1 ± 5.0 points in non-cancer subjects (BCS exercise vs non-cancer subjects: LSMD 0.98; 95% CI: –0.61 to 2.6; *P =* 0.23). SWLS scores increased by 2.5 ± 3.7 points in BCS exercise, 0.7 ± 4.0 points in BCS usual care (LSMD 1.7; 95% CI: 0.43-2.9; *P =* 0.009), and 0.9 ± 4.7 points in non-cancer subjects (BCS exercise vs non-cancer subjects: LSMD 1.7; 95% CI: 0.26-2.2; *P =* 0.021) (Table 2).

## Discussion

These findings provide strong evidence that aerobic exercise significantly improves CRF, subjective vitality, and life satisfaction in long-term BCS (>10 years after diagnosis) compared with usual care. Notably, the CAUSE trial is the first randomized controlled trial in exercise oncology to include similarly aged cancer-naive subjects, offering valuable reference points for interpreting exercise effects. Despite these benefits, the gains in CRF among BCS were less than one-half those observed in non-cancer subjects, indicating a blunted exercise response in BCS (Central Illustration). This attenuated adaptation highlights the long-term physiological burden of cancer treatment and suggests that BCS may require more individualized and extended survivorship care strategies to address persistent treatment-related effects.

**Central Illustration:**
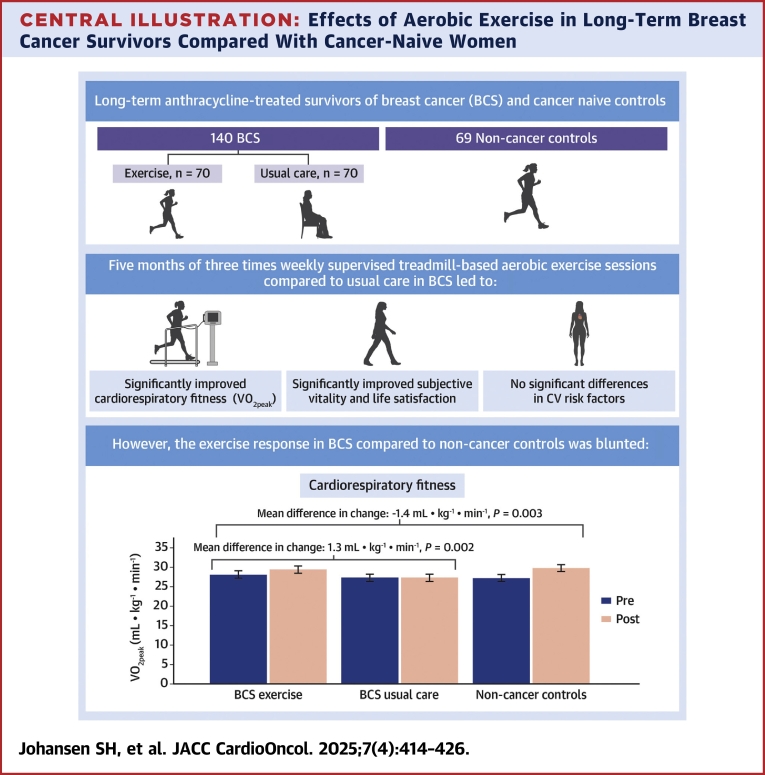
Effects of Aerobic Exercise in Long-Term Breast Cancer Survivors Compared With Cancer-Naive Women This illustration summarizes the effects of a 5-month of aerobic exercise intervention in long-term survivors of breast cancer (BCS), compared with both usual care and non-cancer subjects. Aerobic exercise significantly improved cardiorespiratory fitness (CRF), subjective vitality, and life satisfaction, with no observed effects on cardiovascular risk factors relative to usual care. The graph highlights that the improvements in CRF in BCS were less than one-half those observed in non-cancer subjects, suggesting a blunted exercise response in long-term BCS.

The magnitude of exercise-induced CRF improvement observed in this trial aligns with previous reports in BCS.^16^^,^^28^ For example, Scott et al^16^ reported 0.6 and 0.8 mL O_2_·kg^−1^·min^−1^ after 16 weeks of linear and nonlinear aerobic exercise, respectively, in postmenopausal BCS approximately 3 years after adjuvant therapy. These modest gains, including those in our trial, contrast with the more substantial improvements reported in non-oncological populations. A meta-analysis of 123 trials in apparently healthy individuals found average Vo_2peak_ improvements of 3.9 mL O_2_·kg^−1^·min^−1^ in response to varied aerobic exercise,^29^ whereas typical improvements during or after breast cancer treatment are reported to be around 2.2 mL O_2_·kg^−1^·min^−1^.^15^ These findings support a growing body of evidence pointing to a blunted exercise response in BCS.

The underlying mechanisms for this attenuated response remain under investigation.^30^^,^^31^ Proposed explanations span both central (eg, cardiac output)^32^ and peripheral (eg, arteriovenous oxygen difference [av-O_2_ difference])^33^ components of the cardiopulmonary–muscle axis. Scott et al^30^ found that breast cancer patients had more than twice the risk of abnormal exercise response compared with women at high risk of developing breast cancer, mainly due to impaired oxygen delivery and diffusion. Foulkes et al^32^ further showed that Vo_2peak_ was strongly associated with both cardiac reserve and av-O_2_ difference in breast cancer patients 12 months after completing anthracycline therapy. These studies suggest that the limitations in CRF improvement in BCS are likely multifactorial. However, variability in testing methodologies—such as upright treadmill protocols^30^ vs supine magnetic resonance imaging cycling^32^—introduces differences in cardiovascular loading,^34^ making direct comparisons challenging. Still, understanding these mechanisms is essential for designing personalized exercise interventions that more effectively enhance CRF and overall health in long-term cancer survivors.

Our findings are consistent with previous reports of interindividual heterogeneity in exercise responses among cancer survivors.^16^^,^^17^ In healthy individuals, a 1-mL O_2_·kg^−1^·min^−1^ increase in CRF is associated with a 7% reduction in all-cause mortality—equivalent to a 25% risk reduction per 3.5 mL O_2_·kg^−1^·min^−1^ (1 MET) improvement.^10^^,^^11^ Despite similar exercise adherence across groups, only 17% of BCS in this trial achieved a CRF gain >3.5 mL O_2_·kg^−1^·min^−1^, compared with 33% of non-cancer subjects; 56% of BCS experienced improvements >1 mL O_2_·kg^−1^·min^−1,^ compared with 83% of non-cancer subjects. Notably, these differences persisted in the per-protocol analysis of participants who adhered to more than 70% of the prescribed exercise regimen, suggesting that adherence alone did not account for the disparity in outcomes (Supplemental Section 5, Supplemental Tables 2 to 5).

Moreover, previous studies in sedentary obese adults have shown that higher intensity exercise is associated with a lower proportion of nonresponders.^35^ This raises the possibility that BCS may require a greater exercise dose—either through higher intensity or greater volume—to achieve comparable improvements in CRF.

At baseline, our BCS cohort exhibited well-preserved CRF, in contrast to previous studies.^7^^,^^36^, ^37^, ^38^ A recent meta-analysis examining the effect of systemic anticancer treatment on CRF reported a persistent 18% decline in Vo_2peak_ among long-term cancer survivors (median 8 years post-treatment) compared with non-cancer subjects.^7^ However, the meta-analysis did not differentiate between treatment types, which is an important limitation. For example, Jones et al^36^ reported Vo_2peak_ levels 22% to 33% lower than sedentary norms across 4 cross-sectional breast cancer cohorts, whereas Khouri et al^38^ reported Vo_2peak_ levels 20% lower in doxorubicin-treated BCS approximately 2 years after treatment compared with healthy subjects.

These discrepancies may reflect differences in chemotherapy regimens, particularly the cumulative cardiotoxic dose—typically >250 mg/m^2^ for doxorubicin vs >600 mg/m^2^ for epirubicin.^39^ This possibility is supported by findings from Mijwel et al,^13^ who reported a decline in Vo_2peak_ in the usual care group during epirubicin treatment, with levels returning to baseline by 2-year follow-up.^40^ Together, these data suggest that CRF impairment may be treatment-specific, warranting the need for further research.^7^

Epidemiological studies suggest that post-diagnosis exercise therapy—defined as 150 minutes of moderate physical activity per week—is associated with a 23% adjusted reduction in cardiovascular events and cardiovascular mortality among BCS.^41^ However, evidence regarding its effect on cardiovascular outcomes beyond CRF remains limited.^42^ In our trial, aerobic exercise over 20 weeks did not lead to improvements in cardiovascular risk factors, nor did it produce differences when compared with non-cancer subjects.

By contrast, Dieli-Conwright et al^43^ reported significant reductions in metabolic syndrome z-score—including waist circumference, blood pressure, and serum biomarkers—after 16 weeks of concurrent aerobic and resistance training in sedentary, obese, short-term BCS. The discrepancy may be explained in part by the high baseline prevalence of metabolic syndrome (77%) in that cohort, consistent with findings in non-oncological populations, where exercise benefits are most pronounced in those with elevated baseline cardiovascular risk.^29^ Variations in exercise regimens may explain the differing results, as resistance training has a more pronounced effect on lean body mass, insulin sensitivity, and glucose metabolism than aerobic exercise alone.^44^

The CAUSE trial focused solely on aerobic training, as the primary goal was to improve CRF and evaluate its physiological determinants as secondary and tertiary aims. Included resistance training might have altered the peripheral components of the oxygen cascade, potentially obscuring differences between BCS and non-cancer subjects.

Importantly, aerobic exercise significantly improved both vitality and life satisfaction in long-term BCS. In the general population, low life satisfaction is linked to reduced quality of life and increased anxiety.^45^ Cancer patients often report lower life satisfaction than individuals with other chronic conditions, likely due to the profound impact of cancer on major life events.^46^ At 11 years post-treatment, BCS in the present trial reported slightly lower baseline scores of subjective vitality and life satisfaction compared with non-cancer subjects. However, after 20 weeks of aerobic exercise, BCS showed significant improvements that exceeded the baseline levels of non-cancer subjects, with greater gains in life satisfaction than those observed in subjects.

These findings underscore the importance of targeting life satisfaction in survivorship care, as it is considered one of the most meaningful indicators of quality of life in older adults and clinical populations.^47^ Given the resource-intensive nature of this intervention, personalized rehabilitation strategies are essential—offering structured support for those who require it while allowing others to succeed with minimal guidance.

### Study Limitations

First, the high prerandomization Vo_2peak_ levels among BCS may suggest selection bias, potentially indicating that BCS with poorer CRF were less likely to volunteer. This could affect the external validity of our findings. To minimize selection bias, all eligible BCS were identified through the Cancer Registry of Norway and invited to participate. Second, the study included only BCS who had received epirubicin; those treated with trastuzumab were excluded. Although treatment followed national guidelines consistent with other Western countries, protocols vary by region and cancer subtype, limiting the generalizability of our findings to all BCS. Third, the non-cancer subjects were recruited through advertisements and may represent a more motivated cohort. However, baseline characteristics and exercise adherence were similar between groups.

Additionally, although a 1-year follow-up was initially planned, delays in data collection due to the COVID-19 pandemic prevented completion within the available funding period. We also did not systematically record workload data during CPET for all 210 participants, which precluded analyses of systolic blood pressure–to-workload ratios. Finally, results should be interpreted with caution as no adjustment for multiple testing was performed.

## Conclusions

Aerobic exercise is well tolerated and improves CRF in long-term anthracycline-treated BCS more than a decade after treatment. However, the exercise response was attenuated compared with non-cancer subjects. Although no beneficial effects were observed on cardiovascular risk factors, aerobic exercise significantly enhanced subjective vitality and life satisfaction.

Perspectives **COMPETENCY IN MEDICAL KNOWLEDGE:** Exercise therapy is one of the most effective non-pharmacological strategies for improving overall health in cancer survivors. However, the blunted exercise response observed among long-term BCS highlights the lasting physiological burden of cancer treatment and should be carefully considered when designing exercise prescriptions for this population.

**TRANSLATIONAL OUTLOOK:** Although the mechanisms underlying impaired physiological adaptation and heterogeneous in exercise response among breast cancer survivors require further investigation, exercise therapy remains recommended due to its demonstrated benefits in vitality and life satisfaction.

## Funding Support and Author Disclosures

The study was funded by the Norwegian Cancer Society and Aktiv mot Kreft (Active Against Cancer). Dr Sæter was supported by ProCardio Center for Research-based Innovation, funded by the Norwegian Research Council (#309762). Dr Scott was supported by the Memorial Sloan Kettering Cancer Center Support Grant/Core Grant (P30 CA008748). The authors have reported that they have no relationships relevant to the contents of this paper to disclose.
