# Supplementary material for: Effects of Aerobic Exercise on Cardiorespiratory Fitness and Cardiovascular Risk Factors in Long-Term Breast Cancer Survivors: A Randomized Controlled Trial
Source: JACC CardioOncol. 2025 Jun 17;7(4):414–26. doi: 10.1016/j.jaccao.2025.04.006 (PMC12228135; doi:10.1016/j.jaccao.2025.04.006)
Supplement: Supplementary Material [file mmc1.docx]

**Effects of Aerobic Exercise on Cardiorespiratory Fitness and Cardiovascular Risk Factors in Long-Term Breast Cancer Survivors: A Randomized Controlled Trial**

**Supplemental Materials**

**Supplement 1.** CONSORT Checklist for non-pharmacological trials

**Supplement 2.** Exercise intervention

**Supplement 3.** Data handling

**Supplement 4.** Reasons for missing exercise sessions

**Supplement 5.** Per protocol analysis

**Supplement 6.** References

**Supplement 1.** CONSORT Checklist^4^

| Section/Topic Item | Checklist item no. | CONSORT item | Extension for NPT trials | Page |
| --- | --- | --- | --- | --- |
| Title and abstract |  |  |  |  |
|  | 1a | Identification as a randomized trial in the title |  | Title page |
|  | 1b | Structured summary of trial design, methods, results, and conclusions (for specific guidance see CONSORT for abstracts) | Refer to CONSORT extension for abstracts for NPT trials | 3 |
| Introduction |  |  |  |  |
| Background and objectives | 2a | Scientific background and explanation of rationale |  | 5 |
|  | 2b | Specific objectives or hypotheses |  | 7-9 |
| Methods |  |  |  |  |
| Trial design | 3a | Description of trial design (such as parallel, factorial) including allocation ratio | When applicable, how care providers were allocated to each trial group | 6-7 |
|  | 3b | Important changes to methods after trial commencement (such as eligibility criteria), with reasons |  | NA |
| Participants | 4a | Eligibility criteria for participants | When applicable, eligibility criteria for centers and for care providers | 6 |
|  | 4b | Settings and locations where the data were collected |  | 6 |
| Interventions† | 5 | The interventions for each group with sufficient details to allow replication, including how and when they were actually administered | Precise details of both the experimental treatment and comparator | 7 |
|  | 5a |  | Description of the different components of the interventions and, when applicable, description of the procedure for tailoring the interventions to individual participants. | 7 |
|  | 5b |  | Details of whether and how the interventions were standardized. | 7 |
|  | 5c. |  | Details of whether and how adherence of care providers to the protocol was assessed or enhanced | NA |
|  | 5d |  | Details of whether and how adherence of participants to interventions was assessed or enhanced | 9 |
| Outcomes | 6a | Completely defined pre-specified primary and secondary outcome measures, including how and when they were assessed |  | 7-9 |
|  | 6b | Any changes to trial outcomes after the trial commenced, with reasons |  | NA |
| Sample size | 7a | How sample size was determined | When applicable, details of whether and how the clustering by care providers or centers was addressed | 10 |
|  | 7b | When applicable, explanation of any interim analyses and stopping guidelines |  | NA |
| Randomization: |  |  |  |  |
| - Sequence generation | 8a | Method used to generate the random allocation sequence |  | 7 |
|  | 8b | Type of randomization; details of any restriction (such as blocking and block size) |  | 7 |
| - Allocation concealment mechanism | 9 | Mechanism used to implement the random allocation sequence (such as sequentially numbered containers), describing any steps taken to conceal the sequence until interventions were assigned |  | 7 |
| - Implementation | 10 | Who generated the random allocation sequence, who enrolled participants, and who assigned participants to interventions |  | 7 |
| Blinding | 11a | If done, who was blinded after assignment to interventions (for example, participants, care providers, those assessing outcomes) and how | ~~Whether or not those administering co-interventions were blinded to group assignment~~  If done, who was blinded after assignment to interventions (e.g., participants, care providers, those administering co-interventions, those assessing outcomes) and how | 7 |
|  | 11b | If relevant, description of the similarity of interventions | ~~If blinded, method of blinding and description of the similarity of interventions~~ | NA |
|  | 11c |  | If blinding was not possible, description of any attempts to limit bias | 7 |
| Statistical methods | 12a | Statistical methods used to compare groups for primary and secondary outcomes | When applicable, details of whether and how the clustering by care providers or centers was addressed | 10 |
|  | 12b | Methods for additional analyses, such as subgroup analyses and adjusted analyses |  | 10 |
| Results |  |  |  |  |
| Participant flow (a diagram is strongly recommended) | 13a | For each group, the numbers of participants who were randomly assigned, received intended treatment, and were analyzed for the primary outcome | The number of care providers or centers performing the intervention in each group and the number of patients treated by each care provider or in each center | Figure 1 |
|  | 13b | For each group, losses and exclusions after randomization, together with reasons |  | Figure 1 |
|  | 13c |  | For each group, the delay between randomization and the initiation of the intervention | NA |
|  | new |  | Details of the experimental treatment and comparator as they were implemented | NA |
| Recruitment | 14a | Dates defining the periods of recruitment and follow-up |  | 11 |
|  | 14b | Why the trial ended or was stopped |  | NA |
| Baseline data | 15 | A table showing baseline demographic and clinical characteristics for each group | When applicable, a description of care providers (case volume, qualification, expertise, etc.) and centers (volume) in each group. | Table 1 |
| Numbers analyzed | 16 | For each group, number of participants (denominator) included in each analysis and whether the analysis was by original assigned groups |  | Figure 1 |
| Outcomes and estimation | 17a | For each primary and secondary outcome, results for each group, and the estimated effect size and its precision (such as 95% confidence interval) |  | Table 2 |
|  | 17b | For binary outcomes, presentation of both absolute and relative effect sizes is recommended |  | NA |
| Ancillary analyses | 18 | Results of any other analyses performed, including subgroup analyses and adjusted analyses, distinguishing pre-specified from exploratory |  | Supplemental |
| Harms | 19 | All important harms or unintended effects in each group (for specific guidance see CONSORT for harms) |  | 11 |
| **Discussion** |  |  |  |  |
| Limitations | 20 | Trial limitations, addressing sources of potential bias, imprecision, and, if relevant, multiplicity of analyses | In addition, take into account the choice of the comparator, lack of or partial blinding, and unequal expertise of care providers or centers in each group | 16 |
| Generalizability | 21 | Generalizability (external validity, applicability) of the trial findings | Generalizability (external validity) of the trial findings according to the intervention, comparators, patients, and care providers and centers involved in the trial | 16 |
| Interpretation | 22 | Interpretation consistent with results, balancing benefits and harms, and considering other relevant evidence |  | Discussion |
| Other information |  |  |  |  |
| Registration | 23 | Registration number and name of trial registry |  | 6 |
| Protocol | 24 | Where the full trial protocol can be accessed, if available |  | 6 |
| Funding | 25 | Sources of funding and other support (such as supply of drugs), role of funders |  | Title page |

**Supplement 2. Exercise intervention**

The exercise intervention is described in detail elsewhere.^1^ In brief, the exercise prescription included both continuous sessions at low to moderate intensities and high-intensity intervals (Figure 1).

The continuous sessions were classified into three categories:

- “Black sessions” involved 24-40 minutes at 65% of peak heart rate (HR_peak_) (n=13)
- “Light blue sessions” involved 30 minutes at 75% of HR_peak_ (n=17)
- “Green sessions” involved 30 minutes at 83.5% of HR_peak_ (n=2)

The interval sessions were classified into three categories:

- “Dark blue sessions” included three to five 4-minute intervals at 93.5% of HR_peak_ with 3 minutes of active rest (brisk walking) between intervals (n=16)
- “Purple sessions” included two to four 8-minute intervals at 88% of HR_peak_ with 4 minutes of active rest (brisk walking) between intervals (n=8)
- **** “Orange session” followed the CPET exercise protocol and was performed every fourth week (n=4)

**Supplement Figure 1. Outline of the exercise prescription (adapted from Nilsen et al., 2023^1^)** Bars represent the exercise intensity (% of peak heart rate) at a given exercise session, and the dotted line represents the duration (minutes) at the prescribed exercise intensity in a given session.

**Supplement 3. Data handling**

*Cardiorespiratory fitness*

The gas exchange variables (VO_2_, VCO_2_, VE_peak,_ and RER) represent an average over 30-second intervals, and the highest obtained VO_2_ during the test was registered as VO_2peak_. The ventilatory threshold was calculated using the ventilatory-equivalent method and VE/VCO_2_ slope was calculated using minute ventilation (VE) and VCO_2_ below the respiratory compensation point. Oxygen pulse was calculated by dividing VO_2peak_ (in mL) by the HR_peak_. Maximal effort was defined as RER ≥ 1.10 and lactate ≥7.0 mmol/L in women aged 20 to 49 years, RER ≥ 1.05 and lactate ≥ 5.0 mmol/L in women aged 50 to 64 years, and RER ≥ 1.00 and lactate ≥ 3.5 mmol/L in women ≥ 65 years.^2^ A low CRF level was defined as VO_2peak_ <85% of predicted compared to reference material.^3^

*Cardiometabolic biomarkers*

**Supplemental Table 1**. Biochemical analyses of blood samples

| **Analyze** | **Instrument** | **Method** |
| --- | --- | --- |
| Glucose | Advia Chemistry XPT | Glucose Hexokinase_3 Concentrated (GLUH_c)  Photometry/Colorimetric endpoint |
| HbA1c | Tosoh G8/G11 from 18.10.21 | HPLC (High Performance Liquid Chromatography) |
| Insulin | Advia Centaur XPT | Insulin (IRI)  Chemiluminescence |
| Total cholesterol | Advia Chemistry XPT | Concentrated Cholesterol (CHOL_c),  Photometry/Colorimetric endpoint |
| HDL-C | Advia Chemistry XPT | Direct HDL-cholesterol (D‑HDL),  Photometry/Colorimetric endpoint |
| LDL-C | Advia Chemistry XPT | Direct LDL-cholesterol (DLDL),  Colorimetric endpoint |
| Triglycerides | Advia Chemistry XPT | Triglycerides_2, concentrated (TRIG_c)  Photometry/Colorimetric endpoint |

HbA1c indicates glycated hemoglobin; HDL-C, high-density lipoprotein cholesterol; LDL-C, low-density lipoprotein cholesterol.

*Self-reported physical activity level*

Participants were dichotomized into meeting or not meeting the World Health Organization recommendation of ≥ 150 min of moderate-intensity, ≥ 75 min of vigorous-intensity, or an equivalent combination of moderate and vigorous-intensity physical activity per week.

*Patient-reported outcomes*

Each item on both the Subjective Vitality Scale (SVS) and the Satisfaction With Life Scale (SWLS) was rated on a Likert-scale ranging from (1) “strongly disagree” to (7) “strongly agree”. The total raw score was used for each scale, ranging from 5 to 35, with higher scores indicating greater vitality or life satisfaction. If a participant did not respond to all items on the SVS or SWLS (e.g., answered only 4 of 5 items), the missing item values were imputed by calculating the mean of the completed items within that specific questionnaire for each participant.

**Supplement 4.** Reasons for missing exercise training sessions

|  | **BCSs exercise** | | **Non-cancer controls** | |
| --- | --- | --- | --- | --- |
|  | Participants | Sessions | Participants | Sessions |
| General |  |  |  |  |
| Absence due to general illness* | 41 (59) | 137 | 41 (59) | 156 |
| Musculoskeletal issues | 12 (17) | 25 | 12 (17) | 66 |
| Fracture** | 2 (3) | 25 | 3 (4) | 11 |
| Time Constraints |  |  |  |  |
| Vacation | 47 (67) | 238 | 38 (55) | 193 |
| Unspecified | 20 (29) | 39 | 13 (19) | 34 |
| Work-related | 8 (11) | 37 | 12 (17) | 19 |
| Planned medical appointments | 1 (1) | 2 | 1 (1) | 1 |
| COVID-related |  |  |  |  |
| Diagnosed with COVID | 11 (16) | 44 | 14 (20) | 49 |
| Quarantine | 8 (11) | 10 | 8 (12) | 8 |
| Lock-down | 12 (17) | 20 | 11 (16) | 20 |
| Logistics-related |  |  |  |  |
| Instructor absent | 12 (17) | 47 | 10 (14) | 15 |
| Alternative/ unquantifiable exercise | 12 (17) | 28 | 18 (26) | 48 |
| Cancer-related late-effects |  |  |  |  |
| Anxiety | 1 (1) | 17 | NA | NA |
| Fatigue | 1 (1) | 2 | NA | NA |
| Infected Lymphedema | 1 (1) | 1 | NA | NA |
| Reporting failure |  |  |  |  |
| Missing session without reason | 18 (26) | 74 | 39 (57) | 121 |

Data expressed as number (%). The number of reasons for missing sessions sums to greater than the total number of participants listed because several participants were missing sessions for different reasons. NA indicates not applicable.

* Cold, the flu, headache, etc.

** One study-related

**Supplement 5. Per protocol analysis**

**Supplemental Table 2:** Baseline characteristics of participants included in the per-protocol analysis.

|  | **BCSs Exercise (n=37)** | **BCSs usual care (n=60)** | **Non-cancer controls (n=40)** | ***P* value*** |
| --- | --- | --- | --- | --- |
| ***Socio-demographic*** |  |  |  |  |
| Age at survey, years | 59.5±5.7 | 59.2±7.0 | 57.7±5.0 | 0.15 |
| Living with partner, n (%)  Yes  No | 27 (73.0)  10 (27.0) | 32 (53.3)  14 (23.3) | 29 (72.5)  12 (30.0) | 0.83 |
| Education level >13 years, n (%)  Yes  No | 22 (59.5)  15 (40.5) | 41 (68.3)  19 (31.7) | 29 (72.5)  11 (27.5) | 0.23 |
| ***Lifestyle and health*** |  |  |  |  |
| Height, cm | 168.7±4.9 | 168.1±6.4 | 167.5±6.9 | 0.39 |
| Body mass, kg | 75.8±12.8 | 77.2±14.4 | 76.6±13.4 | 0.79 |
| Body mass index, kg/m^2^ | 26.6±4.11 | 27.3±4.6 | 27.3±4.5 | 0.48 |
| Daily smokers, n (%) | 2 (5.5) | 3 (5.0) | 2 (5.0) | >0.99 |
| Antihypertensive medication, n (%) | 2 (5.4) | 9 (15.0) | 4 (10.0) | 0.68 |
| Lipid-lowering medication, n (%) | 2 (5.4) | 9 (15.0) | 5 (12.5) | 0.44 |
| Glucose-lowering medication, n (%) | 0 (0) | 2 (3.3) | 2 (5.0) | 0.50 |

Data expressed as n (%) or mean±SD. *p*-value is derived from the students t-test for continuous normally distributed variables, Mann-Whitney U test for

continuous non-normally distributed variables, and *χ^2^*-test or Fisher's exact test for categorical variables.

* Comparison between BCSs Exercise and non-cancer controls.

**Supplemental Table 3.** Per-protocol analysis on the effects of aerobic exercise on cardiorespiratory fitness and cardiovascular risk factors in BCSs Exercise compared to BCSs Usual care. The analysis included participants in the exercise group adhering to >70% of the prescribed exercise regimen and participants in the BCSs usual care group with change in self-reported moderate-vigorous physical activity per week <90 minutes from pre- to post-intervention.

|  | **BCSs Exercise** | | | **BCSs Usual Care** | | | **BCSs Exercise vs. BCSs Usual care** |  |
| --- | --- | --- | --- | --- | --- | --- | --- | --- |
|  | n | Pre  (n=37) | Post  (n=37) | n | Pre  (60) | Post  (60) | Least squares mean difference  (95% CI) | *P* value |
| ***Cardiorespiratory fitness*** |  |  |  |  |  |  |  |  |
| VO_2peak_ (L·min) | 37 | 2.05±0.33 | 2.17±0.35 | 60 | 2.05±0.42 | 2.04±0.42 | 0.13 (0.05, 0.21) | 0.002 |
| VO_2peak_ (mL·kg^-1^·min^-1^) | 37 | 27.4±4.9 | 29.3±5.0 | 60 | 26.9±5.1 | 26.9±5.2 | 1.9 (0.9, 3.0) | <0.001 |
| VO_2_ at VT (L·min^-1^) | 36 | 1.68±0.35 | 1.72±0.33 | 59 | 1.70±0.39 | 1.76±0.34 | 0.07 (-0.03, 0.18) | 0.15 |
| HR_peak_ (beats·min^-1^) | 37 | 175±9 | 173±10 | 60 | 173±12 | 172±12 | -0.35 (-2.60, 1.90) | 0.76 |
| O_2_pulse (mL·beat^-1^) | 37 | 11.7±1.87 | 12.6±1.99 | 60 | 11.8±2.50 | 11.8±2.38 | 0.77 (0.30, 1.24) | 0.001 |
| Peak systolic BP (mmHg) | 24 | 201±21 | 199±19 | 35 | 201±18 | 194±24 | 5.5 (-5.2, 16.2) | 0.31 |
| Peak diastolic BP (mmHg) | 24 | 80±19 | 73±15 | 35 | 79±16 | 82±14 | -9.7 (-16.0, -3.4) | 0.003 |
| VE_peak_ (L·min^-1^) | 37 | 78±14 | 87±15 | 60 | 78±17 | 78±17 | 8.2 (4.3, 12.3) | <0.001 |
| VE/VCO_2_ slope | 37 | 28±3 | 29±5 | 42 | 27±4 | 28±4 | 0.40 (-0.94, 1.73) | 0.56 |
| SpO_2_ at max effort (%) | 33 | 95±3 | 95±3 | 53 | 93±3 | 94±2 | -0.62 (-1.39, 1.15) | 0.11 |
| RER (VCO_2_·VO2^-1^) | 37 | 1.20±0.09 | 1.21±0.07 | 60 | 1.20±0.09 | 1.19±0.07 | 0.02 (0.00, 0.04) | 0.042 |
| Blood lactate (mmol) | 31 | 8.20±2.60 | 8.36±2.60 | 53 | 8.50±2.39 | 8.02±2.32 | 0.56 (-0.16, 1.28) | 0.13 |
| Borgs scale_6-20_ | 35 | 17±1 | 18±1 | 53 | 17±2 | 17±2 | 0.83 (0.38, 1.28) | <0.001 |
| ***Cardiovascular risk factors*** |  |  |  |  |  |  |  |  |
| Self-reported moderate to vigorous physical activity, (minutes/week) | 37 | 93.5±97.6 | 145.1±89.3 | 60 | 102.6±162.5 | 88.6±141.7 | 63.3 (35.6, 91.1) | <0.001 |
| Systolic blood BP (mmHg) | 37 | 137±18 | 136±18 | 60 | 134±18 | 135±17 | -1.30 (-4.62, 2.02) | 0.44 |
| Diastolic BP (mmHg) | 37 | 84±8 | 82±10 | 60 | 82±8 | 81±8 | -1.65 (-4.03, 0.73) | 0.17 |
| Fasted glucose (mmol·L^-1^) | 36 | 5.15±0.59 | 5.10±0.65 | 55 | 5.20±0.9 | 5.21±1.04 | -0.11 (-0.31, 0.10) | 0.29 |
| HbA1c (mmol·mol^-1^) | 36 | 36.6±3.43 | 37.0±3.13 | 55 | 38.2±4.3 | 37.8±4.62 | 0.76 (0.19, 1.33) | 0.009 |
| Insulin (pmol·L^-1^) | 36 | 53.4±24.4 | 47.2±20.9 | 55 | 59.5±50.6 | 57.4±48.4 | -6.09 (-17.59, 5.41) | 0.30 |
| Total cholesterol (mmol·L^-1^) | 36 | 5.97±0.98 | 5.84±0.98 | 55 | 6.00±0.89 | 5.91±0.88 | -0.04 (-0.28, 1.97) | 0.72 |
| HDL-C (mmol·L^-1^) | 36 | 1.81±0.48 | 1.80±0.45 | 55 | 1.83±0.44 | 1.79±0.43 | 0.03 (-0.06, 0.12) | 0.52 |
| LDL-C (mmol·L^-1^) | 36 | 3.91±0.96 | 3.70±0.91 | 55 | 3.97±0.89 | 3.93±0.90 | -0.19 (-0.45, 0.08) | 0.17 |
| Triglycerides (mmol·L^-1^) | 36 | 1.15±0.51 | 1.12±0.38 | 55 | 1.25±0.64 | 1.25±0.73 | -0.05 (-0.21, 0.11) | 0.51 |
| MicroCRP (mg·L^-1^) | 36 | 1.93±2.68 | 1.84±1.99 | 55 | 1.68±1.59 | 2.03±3.96 | -0.33 (-1.67, 1.01) | 0.63 |
| Total fat mass (kg) | 37 | 31.2±8.8 | 30.0±8.4 | 60 | 31.9±9.6 | 31.6±9.4 | -0.8 (-1.7, -0.1) | 0.033 |
| Fat percentage (%) | 37 | 40±5 | 39±5 | 60 | 41±5 | 40±5 | -0.8 (-1.5, -0.1) | 0.020 |
| Total lean body mass (kg) | 37 | 42.1±4.8 | 42.3±4.6 | 60 | 42.6±5.6 | 42.6±5.4 | 0.1 (-0.3, 0.5) | 0.70 |

Data expressed as mean±SD. *p*-value is derived from an analysis of covariance (ANCOVA). VO_2peak_ indicates peak oxygen consumption; VT, ventilatory threshold; HR_peak_, peak heart rate; O_2_pulse, oxygen pulse; BP, blood pressure; VE_peak_, peak minute ventilation; VE/VCO_2_, ventilatory equivalent for carbon dioxide output; SpO_2_, oxygen saturation; RER, respiratory exchange ratio; mmHg, millimeters of mercury; HbA1c, glycated hemoglobin; HDL-C, high-density lipoprotein cholesterol; LDL-C, low-density lipoprotein cholesterol; CRP, C-reactive protein.

^a^ Analyses were adjusted for baseline values of the respective outcome variable

**Supplemental Table 4**. Per-protocol analysis on effects of aerobic exercise on change in cardiorespiratory fitness and cardiovascular risk factors between participants in BCSs Exercise and non-cancer controls adhering to >70% of the prescribed exercise regimen.

|  | **Exercise response** | | | | **BCSs exercise vs Non-cancer controls^a^** |  |
| --- | --- | --- | --- | --- | --- | --- |
|  | n | BCSs Exercise (n=37) | n | Non-cancer controls (n=40) | Least squares mean difference  (95% CI) | *P* value |
| ***Cardiorespiratory fitness*** |  |  |  |  |  |  |
| VO_2peak_ (L·min) | 37 | 0.12±0.21 | 40 | 0.21±0.17 | -0.08 (-0.17, 0.00) | 0.053 |
| VO_2peak_ (mL·kg^-1^·min^-1^) | 37 | 1.9±2.6 | 40 | 3.3±2.5 | -1.2 (-2.3, -0.4) | 0.043 |
| VO_2_ at VT (L·min^-1^) | 36 | 0.04±0.34 | 39 | 0.11±0.20 | -0.07 (-0.19, 0.06) | 0.31 |
| HR_peak_ (beats·min^-1^) | 37 | -1.92±6.89 | 40 | -1.92±7.64 | 0.32 (-3.08, 3.71) | 0.85 |
| O_2_pulse (mL·beat^-1^) | 37 | 0.83±1.10 | 40 | 1.37±1.04 | -0.52 (-1.00, -0.04) | 0.035 |
| Peak systolic BP (mmHg) | 24 | -1.5±22.2 | 22 | -5.1±34.2 | 4.5 (-13.6, 22.6) | 0.62 |
| Peak diastolic BP (mmHg) | 24 | -7.1±17.9 | 22 | 6.0±19.0 | -16.7 (-27.9, -5.5) | 0.004 |
| VE_peak_ (L·min^-1^) | 37 | 8±12 | 40 | 6±7 | 3.0 (-1.7, 7.6) | 0.20 |
| VE/VCO_2_ slope | 37 | 0.9±4.0 | 39 | -0.6±3.2 | 1.6 (-1.2, 3.3) | 0.08 |
| SpO_2_ at max effort (%) | 30 | -0.7±2.0 | 25 | -0.4±2.3 | -0.4 (-1.8, 0.9) | 0.52 |
| RER (VCO_2_·VO2^-1^) | 37 | 0.01±0.07 | 40 | -0.01±0.07 | 0.03 (-0.01; 0.06) | 0.11 |
| Blood lactate (mmol) | 31 | 0.21±1.98 | 34 | -0.22±1.75 | 0.44 (-0.53, 1.40) | 0.37 |
| Borgs scale_6-20_ | 35 | 0.86±1.64 | 35 | 0.86±1.19 | 0.02 (-0.69, 0.73) | 0.95 |
| ***Cardiovascular risk factors*** |  |  |  |  |  |  |
| Self-reported moderate to vigorous physical activity, minutes | 37 | 51.6±71.3 | 40 | 71.1±173,9 | -16.7 (-57.9, 24.5) | 0.42 |
| Systolic BP (mmHg) | 37 | -0.59±8.29 | 40 | -1.44±12.06 | 1.13 (-3.60, 5.86) | 0.48 |
| Diastolic BP (mmHg) | 37 | -2.22±6.21 | 40 | -2.00±6.26 | 0.28 (-2.54, 3.09) | 0.85 |
| Fasted glucose (mmol·L^-1^) | 36 | -0.06±0.47 | 32 | -0.04±0.41 | -0.08 (-0.31, 0.15) | 0.51 |
| HbA1c (mmol·mol^-1^) | 36 | 0.48±1.37 | 38 | 0.32±2.00 | 0.36 (-0.47, 1.19) | 0.39 |
| Insulin (pmol·L^-1^) | 18 | -12.06±20.7 | 31 | 3.32±21.07 | -16.1 (-28.29, -3.90) | 0.011 |
| Total cholesterol (mmol·L^-1^) | 36 | -1.14±0.79 | 38 | -0.31±0.63 | 0.19 (-0.16, 0.54) | 0.27 |
| HDL-C (mmol·L^-1^) | 36 | -0.01±0.17 | 38 | 0.02±0.25 | -0.04 (-0.14, 0.07) | 0.47 |
| LDL-C (mmol·L^-1^) | 36 | -0.21±0.97 | 38 | -0.19±0.60 | 0.06 (-0.32, 0.45) | 0.74 |
| Triglycerides (mmol·L^-1^) | 36 | -0.04±0.34 | 38 | -0.10±0.32 | 0.07 (-0.08, 0.23) | 0.35 |
| MicroCRP (mg·L^-1^) | 36 | -0.21±1.83 | 35 | 0.27±3.30 | -0.56 (-1.91, 0.79) | 0.41 |
| Total fat mass (kg) | 37 | -1.14±2.30 | 39 | 1.37±2.30 | 0.22 (-0.79, 1.24) | 0.66 |
| Fat percentage (%) | 37 | -1.00±2.05 | 39 | -1.06±1.71 | 0.17 (-0.71, 1.05) | 0.70 |
| Total lean mass (kg) | 37 | 0.13±1.16 | 39 | 0.30±1.10 | -0.28 (-0.78, 0.23) | 0.29 |

Data expressed as mean±SD. *p*-value is derived from an analysis of covariance (ANCOVA). VO_2peak_ indicates peak oxygen consumption; VT, ventilatory threshold; HR_peak_, peak heart rate; O_2_pulse, oxygen pulse; BP, blood pressure; VE_peak_, peak minute ventilation; VE/VCO_2_, ventilatory equivalent for carbon dioxide output; SpO_2_, oxygen saturation; RER, respiratory exchange ratio; mmHg, millimeters of mercury; HbA1c, glycated hemoglobin; HDL-C, high-density lipoprotein cholesterol; LDL-C, low-density lipoprotein cholesterol; CRP, C-reactive protein.

^a^ Analyses were adjusted for age, BMI and self-reported moderate-vigorous physical activity per week at baseline

**Supplemental Table 5.** Per-protocol analysis on the change in Subjective Vitality Scale and Satisfaction With Life Scale in participants in BCSs. The analysis included participants in the exercise groups adhering to >70% of the prescribed exercise regimen and participants in the BCSs usual care group with change in self-reported moderate-vigorous physical activity per week <90 minutes from pre- to post-intervention.

|  | **Pre** | |  | **Post** | |  | **BCSs Exercise vs Usual care^a^** | | **BCSs Exercise vs non-cancer controls^b^** | |
| --- | --- | --- | --- | --- | --- | --- | --- | --- | --- | --- |
|  | BCSs Exercise  (n=37) | BCSs Usual care  (n=60) | Non-cancer controls (n=40) | BCSs Exercise  (n=37) | BCSs Usual care  (n=60) | Non-cancer controls (n=40) | Least squares mean difference  (95% CI) | *P* value | Least squares mean difference  (95% CI) | *P* value |
| ***SVS total score*** | 24.7±6.5 | 24.0±6.4 | 27.1±5.4 | 28.1±5.7 | 24.0±5.8 | 28.9±6.2 | 3.5 (1.8, 5.1) | <0.001 | 2.7 (0.4, 5.1) | 0.024 |
| ***SWLS total score*** | 26.1±4.8 | 25.5±5.6 | 27.8±5.3 | 28.5±5.2 | 25.9±5.9 | 28.7±5.03 | 1.9 (0.5, 3.1) | 0.010 | 2.4 (0.4, 4.3) | 0.019 |

Data expressed as mean (SD). *p*-value is derived from an analysis of covariance (ANCOVA). SVS indicates Subjective Vitality Scale; SWLS Satisfaction With Life Scale.

^a^ Analyses were adjusted for baseline values of the respective outcome variable

^b^ Analyses were adjusted for age, BMI and self-reported moderate-vigorous physical activity per week at baseline

**Supplement 6. REFERENCES**

1. Nilsen TS, Sæter M, Sarvari SI, Reinertsen KV, Johansen SH, Edvardsen ER, et al. Effects of Aerobic Exercise on Cardiorespiratory Fitness, Cardiovascular Risk Factors, and Patient-Reported Outcomes in Long-Term Breast Cancer Survivors: Protocol for a Randomized Controlled Trial. JMIR Res Protoc. 2023;12:e45244.

2. Edvardsen E, Hem E, Anderssen SA. End Criteria for Reaching Maximal Oxygen Uptake Must Be Strict and Adjusted to Sex and Age: A Cross-Sectional Study. PLOS ONE. 2014;9(1):e85276.

3. Edvardsen E, Hansen BH, Holme IM, Dyrstad SM, Anderssen SA. Reference Values for Cardiorespiratory Response and Fitness on the Treadmill in a 20- to 85-Year-Old Population. Chest. 2013;144(1):241–8.

4. Boutron I, Altman DG, Moher D, Schulz KF, Ravaud P, CONSORT NPT Group. CONSORT Statement for Randomized Trials of Nonpharmacologic Treatments: A 2017 Update and a CONSORT Extension for Nonpharmacologic Trial Abstracts. Ann Intern Med. 2017;167(1):40–7.
